# Encephalitis Associated to Metabotropic Glutamate Receptor 5 (mGluR5) Antibodies in Cerebrospinal Fluid

**DOI:** 10.3389/fimmu.2018.02568

**Published:** 2018-11-05

**Authors:** Carlos Guevara, Gonzalo Farias, Carlos Silva-Rosas, Pablo Alarcon, Gabriel Abudinen, Julio Espinoza, Andrés Caro, Heather Angus-Leppan, Jose de Grazia

**Affiliations:** ^1^Faculty of Medicine, Universidad de Chile, Santiago, Chile; ^2^Clinical Neurosciences, Royal Free London NHS Foundation Trust, London, United Kingdom; ^3^Institute of Neurology, University College London, London, United Kingdom; ^4^Centre for Research in Public Health and Community Care, University of Hertfordshire, Hertfordshire, United Kingdom

**Keywords:** encephalitis, metabotropic glutamate receptor 5, Hodgkin's lymphoma, Ophelia syndrome, limbic encephalitis

## Abstract

A 68-years-old Hispanic man, complained of night sweats, low grade fewer, unexplained weight loss, and memory problems over 3 months. Abdominal tomography showed multiple intra-abdominal adenopathy and biopsy confirmed classic Hodgkin's lymphoma. He commenced treatment with chemotherapy. Three months later, he had acute onset of inattention, auditory hallucinations and alterations of anterograde memory. The patient developed psychomotor agitation, unresponsive to a combination of neuroleptics and benzodiazepines. Brain MRI showed a small established cerebellar infarction. Electroencephalogram was normal. Tests for toxic metabolic encephalopathy were negative. One oligoclonal IgG bands was found in the Cerebrospinal fluid (CSF), which was not observed in corresponding serum, but cell count and protein were normal. Extensive testing for infectious encephalitis was unremarkable. CSF testing for commercially available neural and non-neural autoantibodies was negative. The patient fulfilled the Gultekin diagnostic criteria for paraneoplastic limbic encephalitis and methylprednisolone IV 1g/d for 5 days was given. He recovered rapidly, with progressive improvement in memory and psychomotor agitation. After treatment commenced, results for antibodies to mGluR5 in CSF taken prior to treatment were returned as positive. mGluR5 is found on post-synaptic terminals of neurons and microglia and is expressed primarily in the hippocampus and amygdala. This case highlights the difficulties in diagnosing this type of encephalitis: the CSF did not show pleocytosis, the MRI showed only chronic change and the electroencephalogram was normal. The dramatic recovery after methylprednisolone help to better characterized the clinical spectrum of auto-immune encephalitis. Diagnosing anti mGlutR5 encephalitis may lead to potentially highly effective treatment option and may anticipate the diagnostic of a cancer. A high index of suspicion is needed to avoid missed diagnosis. In patients with unexplained encephalitis, testing for antibodies to mGluR5 in CSF and serum should be considered. When there is a reasonable index of suspicion of auto-immune encephalitis, treatment should not be delayed for the antibody results.

## Clinical case

A 68-years-old Hispanic man with chronic depression and anxiety, complained of night sweats, low grade fewer, and unexplained weight loss over 3 months. He had also 3 months of difficulty managing finances and keeping track of appointments. Abdominal tomography showed multiple intra-abdominal adenopathy and biopsy confirmed classic Hodgkin's lymphoma, of nodular sclerotic variety. He commenced treatment with ABVD chemotherapy (adriamycin, vinblastine, bleomycin, and dacarbazine). Six months after first presentation of the cognitive problems, he had acute onset of disorientation, inattention, psychomotor agitation, confusion, delusional ideas of grandiosity, auditory hallucinations, and alterations of anterograde memory. His score was 20/30 on the Montreal Cognitive Assessment (MoCA) suggesting severe cognitive impairment. Two days later, the patient developed multiple episodes of psychomotor agitation and was unresponsive to a combination of neuroleptics and benzodiazepines. These neuropsychiatric changes were not attributed to the ongoing stable treatment with ABVD chemotherapy. Brain MRI showed a small established cerebellar infarction. Electroencephalogram was normal. Tests for metabolic encephalopathies were negative: complete blood cell count, calcium, magnesium, phosphorus, liver function tests, erythrocyte sedimentation rate, antinuclear antibody, C-reactive protein, thyroid-stimulating hormone, antithyroglobulin, antithyroperoxidase antibodies, cortisol, vitamin B_12_, and laboratory tests for toxicology. Human immunodeficiency virus and rapid plasma reagin were negative. One oligoclonal IgG bands was found in the CSF, which was not observed in corresponding serum, but cell count and proteins were normal. CSF Gram stain and culture were negative. Extensive testing for infectious encephalitis was unremarkable (CSF PCR for *E. coli K1, H. influenzae, L. monocytogenes, N. meningitidis, S. agalactiae, S. pneumoniae, Cytomegalovirus, Enterovirus, Herpes simplex 1* and *2, Herpes 6, Parechovirus, Varicella zoster*, and *Cryptococcus neoformans*). The patient fulfilled the diagnostic criteria by Gultekin et al. for paraneoplastic limbic encephalitis (PLE) (1) and methylprednisolone one gram daily for 5 days was given. The patient recovered rapidly, with progressive improvement in memory and psychomotor agitation. CSF testing for commercially available neural and non-neural autoantibodies was negative (including against the N-Methyl-D-aspartate (NMDA) receptor, AMPA (α-amino-3-hydroxy-5-methyl-4-isoxazolepropionic acid) receptor, and VGKC (voltage-gated potassium channel complex). No informative autoantibodies were detected in the CSF paraneoplastic evaluation. AGNA-1 [Anti-Glial Nuclear antibody (Ab)], Amphiphysin Ab, ANNA-1, 2 and 3 (antineuronal nuclear Ab), CRMP-5-IgG (Collapsin response-mediator protein-5), PCA-1, 2, and 3 (Purkinje Cell Cytoplasmic Ab). Further testing of CSF for antibodies to metabotropic glutamate receptor 5 (mGluR5) was positive on cell based assay and immunohistochemistry (2). This CSF sample was drawn before starting systemic steroids. At 30-days follow-up, the patient evolved oriented, attentive, without psychomotor agitation. MoCA was 30/30. He remains amnesic with respect to the hospitalization period, but with conservation of other memory modalities. Follow-up CT scan and EEG were unremarkable.

## Background

Glutamate is the major excitatory neurotransmitter in the central nervous system and glutamatergic neurotransmission is involved in most aspects of normal brain function. Dysfunction of glutamate receptors have recently been related to immune-mediated encephalitis (3, 4). The metabotropic glutamate receptors belong to a family of G protein-coupled receptors that have been divided into three groups based on their sequence homology, putative signal transduction mechanisms, and pharmacologic properties (3, 4). mGluR1 and mGluR5 constitute Group I metabotropic glutamate receptors. mGluR5 is found on post-synaptic terminals of neurons and microglia. mGluR5 signals via Gq/G11 coupling to activate phospholipase C, resulting in calcium mobilization and activation of protein kinase C, and are expressed primarily in the hippocampus and amygdala. The antibodies cause a decrease of mGluR5 cluster density at both synaptic and extrasynaptic locations (2), although the exact mechanism by which the antibodies alter the receptor density is unknown. Their location may explain the typical behavioral and memory problems in this mGluR5 antibody—associated encephalitis. Clinical correlates of mGluR5 antibodies have been reported in only 11 patients (2).

## Discussion

In the case of encephalitis with mGluR5 antibodies, Ophelia syndrome (neuropsychiatric abnormalities and coexisting Hodgkin's lymphoma) improvement with steroids is common (2). The resulting neuropsychiatric abnormalities can be diverse, ranging from mood and personality changes to anterograde amnesia, disorientation, headaches, involuntary movements, and fatigue (5, 6).

The diagnosis of anti-mGluR5 encephalitis is rare, but may increase as antibody testing become more widely available. The close link between the autoimmune response and Hodgkin's lymphoma or other malignancies may also contribute to its under-recognition, as neuropsychiatric changes may be attributed to treatment or psychological factors (2, 3, 5–7).

However, anti-mGluR5 encephalitis can occur without tumor: in the eleven previously reported cases of encephalitis with mGluR5 (2), the association with tumors occurred in six patients: five had Hodgkin's lymphoma and one had small cell lung cancer. In all five patients with Hodgkin's lymphoma, the neuropsychiatric disorders preceded the tumor diagnosis by 2–11 months (2), which also occurred in the present case.

This patient satisfied diagnostic criteria for PLE as he had intrathecal oligoclonal IgG band. Following the Gultekin criteria (1), we suggest that patients with unexplained psychiatric disorders should be tested for anti-mGluR5 encephalitis when there is at least one of the following abnormalities: CSF showing inflammatory changes (pleocytosis, oligoclonal bands, increased immunoglobulin content or increased protein content in the absence of measured immunoglobulin); MRI showing unilateral or bilateral temporal lobe abnormalities on T_2_ weighted images and EEG showing slow-or sharp-wave activity in one or both temporal lobes. Antibody status is not essential for early diagnosis and early treatment of autoimmune encephalitis should not be delayed, as antibody testing is not readily accessible in many institutions and result can take several weeks to obtain. The initial diagnostic approach should be based on neurological assessment and tests that are accessible to most clinicians (8). This does not imply that any manifestation of psychiatric disease should give occasion to testing for antineural antibodies, but that it should be done when clinically indicated.

This case highlights the difficulties in diagnosing auto-immune encephalitis as the paucity of positive diagnostic tests may have delayed or prevent the diagnosis of PLE. In this case, the MRI showed only chronic non-specific change. In the recent series of Spatola (2), six out eleven patients (55%) had normal MRI. Gultekin et al. (1) reported that 16 of 44 (36%) patients with PLE had a normal MRI signal. This patient had a normal EEG. Fifty percent of the patients reported by Spatola had also a normal EEG (2). CSF did not show pleocytosis, but all the 11 previous patients reported with antibodies to mGluR5 had pleocytosis (2). Whether this negative finding will be included in the clinical spectrum of this encephalitis or whether is related to a false-positive result for mGluR5 antibody testing and/or the possibility of epiphenom should be assessed after new publications of case reports and case series of this type of encephalitis. Thus, encephalitis with mGluR5 antibodies, along with other autoimmune encephalitis, may present with memory or behavioral deficits with non-diagnostic CSF, imaging and EEG.

This patient had an excellent response to treatment, which should not be delayed awaiting the antibody results.

## Concluding remarks

Diagnosing anti mGluR5 encephalitis is relevant and important, as it may lead to potentially highly effective treatment option and sometimes anticipates the diagnostic of a cancer. In patients with unexplained encephalitis, CSF and serum testing for antibodies to mGluR5 should be considered. Treatment should not be delayed for return of the antibodies when a clinical diagnosis of auto-immune encephalitis is made.

## Ethics statement

The study was conducted according to International Standards of Good Clinical Practice (ICH guidelines and the Declaration of Helsinki). The project was approved by the local Research Ethics Committees of Universidad de Chile Hospital, Santiago, Chile.

## Author contributions

CG: conception, organization, execution, review, and critique; GF, CS, GA, PA, JE, AC, HA-L, and JdG: review and critique.

### Conflict of interest statement

The authors declare that the research was conducted in the absence of any commercial or financial relationships that could be construed as a potential conflict of interest.
